# Divergent trends in Alzheimer’s disease burden: a comparative analysis of the US and G20 nations

**DOI:** 10.3389/ijph.2026.1609525

**Published:** 2026-08-05

**Authors:** Yuexiao Zou, Qingxian Wang, Qingxiang Zhang, Xiaorui Cheng

**Affiliations:** Shandong University of Traditional Chinese Medicine, Jinan, China

**Keywords:** Alzheimer’s disease and other dementias, forecasting models, global burden of disease, Joinpoint regression, risk factors

## Abstract

**Objective:**

Alzheimer’s disease and other dementias (ADOD) impose growing systemic stress. This study compared epidemiological trends and risk factors between the US and G20 to guide resource allocation.

**Methods:**

Using GBD 2023 data, we analyzed incidence, mortality, and DALY rates with Joinpoint regression (1990–2023) and ARIMA forecasting (to 2040).

**Results:**

In 2023, US age-standardized incidence rate was 130 (95% CI: 110.5–147.3), mortality rate 30 (7.4–73.2), and DALY rate 510 (224.8–1059.6), all declining from 1990. G20 rates remained lower but showed an upward inflection after 2020. Joinpoint analysis revealed overall downward trends, with a transient US resurgence (1998–2004); projections suggest continued deceleration. Smoking and high fasting plasma glucose burdens in the US were disproportionately elevated compared to G20.

**Conclusion:**

The US achieved morbidity compression yet faced rising absolute caseloads due to demographic momentum. G20’s post-2020 upturn signals an emerging burden. A shift from smoking to metabolic drivers necessitates primary prevention for G20 and targeted metabolic management for the US.

## Introduction

Alzheimer’s disease and other dementias (ADOD), among the most prevalent neurodegenerative disorders, presents a pressing and growing challenge to public health worldwide [1]. Accelerating population ageing has driven substantial increases in the prevalence, mortality, and overall burden of ADOD and related dementias, placing considerable strain on health systems, social care infrastructure, and household economies. Current estimates suggest approximately 57 million individuals are living with dementia globally [2], the majority of whom have Alzheimer’s disease. Notably, age-standardised incidence rates are declining in many high-income settings, yet continue to rise across low- and middle-income countries—a divergence that underscores marked international and regional inequalities.

Recent evidence further illuminates the complex array of factors contributing to these geographical disparities. In addition to population ageing, modifiable lifestyle and metabolic risks—such as smoking and elevated fasting plasma glucose—are recognised as important determinants of both the onset and progression of ADOD [3, 4]. Some cases of dementia can be prevented or delayed by addressing key risk factors that contribute to it [5]. While a number of studies have examined the global or regional epidemiology of ADOD, most have focused on particular geographical areas such as Europe or Asia. There remains a lack of systematic comparison between the United States and the collective G20—groups that differ substantially in socioeconomic profiles and health system capacities [2, 6]. Moreover, existing analyses have largely been descriptive in nature, seldom employing segmented regression or time-series forecasting approaches to analyse long-term trends and project future trajectories. This limits their utility in shaping forward-looking, evidence-based health policy.

To address these gaps, this study draws on the most recent Global Burden of Disease (GBD) data to analyse trends in ADOD incidence, mortality, and disability-adjusted life years (DALYs) in the United States and G20 countries between 1990 and 2023. The G20 (Group of Twenty), comprising 19 sovereign nations (Argentina, Australia, Brazil, Canada, China, France, Germany, India, Indonesia, Italy, Japan, South Korea, Mexico, Russia, Saudi Arabia, South Africa, Turkey, the United Kingdom, and the United States) and the European Union, represents a diverse group of global economies. We employ segmented regression and time-series forecasting to analyze long-term trends and project future trajectories. In addition, we assess changes in the proportion of ADOD burden attributable to two modifiable risk factors: high fasting plasma glucose and smoking. Together, these analyses are intended to inform more differentiated and precisely targeted strategies for ADOD prevention and control across varied national contexts.

## Methods

### Data source

GBD 2023 quantified the burden of 375 diseases and injuries and 292 causes of death, alongside 88 risk factors categorized into environmental, occupational, behavioral, and metabolic clusters [7]. A detailed elucidation of the general methodology and estimation procedures employed in GBD 2023 is available in previous literature [8]. The G20 aggregate data used in this study were retrieved from the GBD 2023 pre-aggregated region estimates, which include the United States. The US was independently extracted as a standalone comparator. We explicitly acknowledge this compositional overlap in the interpretation of our results.

### Risk factors

We interrogated the Global Health Data Exchange (GHDx) to extract data regarding potential risk antecedents. Leveraging the comparative risk assessment (CRA) framework of GBD 2023, we quantified the disease burden attributable to two distinct ADOD risk factors: a metabolic driver (high fasting plasma glucose) and a behavioral determinant (smoking). The methodology used to apportion the proportional burden of these risk factors has been rigorously detailed in prior investigations [7]. Specifically, this analysis evaluated the percentage contribution of these factors to the ADOD-related DALYs within G20 for the reference years 1990 and 2023.

### Statistical analysis

We abstracted key epidemiological metrics—incidence, mortality, and disability-adjusted life years (DALYs)—along with their corresponding age-standardized rates (ASIR, ASMR, and ASDR) for the United States and G20 from the GBD 2023 dataset. All estimates were reported with 95% uncertainty intervals (UIs).

To delineate significant shifts in long-term trends, we employed Joinpoint regression models to calculate the annual percent change (APC) and average annual percent change (AAPC), accompanied by 95% confidence intervals (CIs). This modeling approach establishes a segmented regression based on the temporal characteristics of disease distribution, dividing the study period into distinct intervals for trend fitting and optimization. This method effectively mitigates the subjectivity inherent in conventional linear trend analyzes. Trend directionality was determined via the AAPC: a 95% CI above zero indicates an increasing trend, a 95% CI below zero denotes a decreasing trend, and an interval crossing zero implies stability.

Furthermore, we utilized (ARIMA) models to forecast ADOD incidence, mortality, and DALYs for the US and G20 through the 2024–2040 horizon. As a robust econometric tool, ARIMA is adept at analyzing the behavior of both stationary and non-stationary time series, allowing for the assessment of how planning and policy interventions may impact specific outcomes over time [9]. In the ARIMA (p, d, q) structure, ‘p' denotes the number of autoregressive terms, ‘d' the degree of differencing, and ‘q' the number of moving average terms [9]. Computational protocols aligned with those reported in previous studies. All statistical analyzes and visualizations were executed using R software (version 4.2.1) (https://cran.r-project.org/src/base/R-4/) and the Joinpoint Regression Program (version 5.2.0) [10]. A two-sided p-value <0.05 was considered statistically significant.

## Results

Regarding absolute disease burden, the United States faced a substantial escalation between 1990 and 2023. Data indicate that the number of incident ADOD cases in the US surged from a baseline of 468,563 (95% UI: 400,540–529,658) in 1990 to 838,746 (95% UI: 706,997–950,122) in 2023, representing an overall increase of 79.00%. The mortality burden intensified even more acutely, exhibiting a 104.72% rise over the same period, culminating in 202,000 deaths in 2023. Similarly, DALYs accumulated markedly, rising from 1.756 million in 1990 to 3.361 million in 2023 (a 91.45% increase). In parallel, the G20 aggregate experienced a more pronounced surge, with incident cases rising from approximately 2.72 million in 1990 to 8.11 million in 2023 (a 198% increase), and DALYs escalating from 9.84 million to 30.20 million (a 207% increase).

However, age-standardization unveiled a divergent epidemiological profile. While absolute numbers climbed, the ASIR and ASDR in the US actually demonstrated statistically significant downward trends over the past three decades, recording AAPCs of −0.2 (95% CI: -0.2, −0.2) and −0.08 (95% CI: -0.09, −0.07), respectively. Meanwhile, changes in ASMR lacked statistical significance (AAPC: -0.003, 95% CI: -0.02, 0.02), pointing to a relative stabilization in mortality rates. In a cross-sectional comparison, although current US values for ASIR, ASMR, and ASDR remain elevated above the G20 average, the longitudinal trajectories reveal a fundamental distinction: the US has entered a plateau or decline phase in standardized rates, whereas the trend across G20 is generally on an upward trajectory (Table 1).

**TABLE 1:** Alzheimer’s disease and other dementias cases, age-standardized incidence, mortality, and disability-adjusted life years rates, with corresponding average annual percent changes (Global Burden of Disease Study 2021, United States and G20 countries, 1990–2023).

Location	Measure	1990	​	2023	​	​
​	​	All-ages cases	ASR	All-ages cases	ASR	1990–2023 AAPC
​	​	n	n	n	n	n
USA	Deaths	98,915.7	30.0	202,495.6	30.0	−0.003
	DALYs	1,755,721.8	526.1	3,361,271.5	510.0	−0.08
	Incidence	468,563.3	138.2	838,745.6	130 .0	−0.2
G20	Deaths	488,156	25.9	1,677,443.0	26.9	0.2
	DALYs	9,844,871.7	459.2	30,204,890.3	475.2	0.1
	Incidence	2,718,567.0	118.9	8,105,144.4	126.3	0.2

* USA United States of America, G20 Group of 20, UI uncertainty interval, CI confidence interval, DALYs disability-adjusted life years, AAPC average annual percentage change, ASR Age-standardized rates (per 100,000 people).

### Overall trends and Joinpoint analysis

**FIGURE 1:**
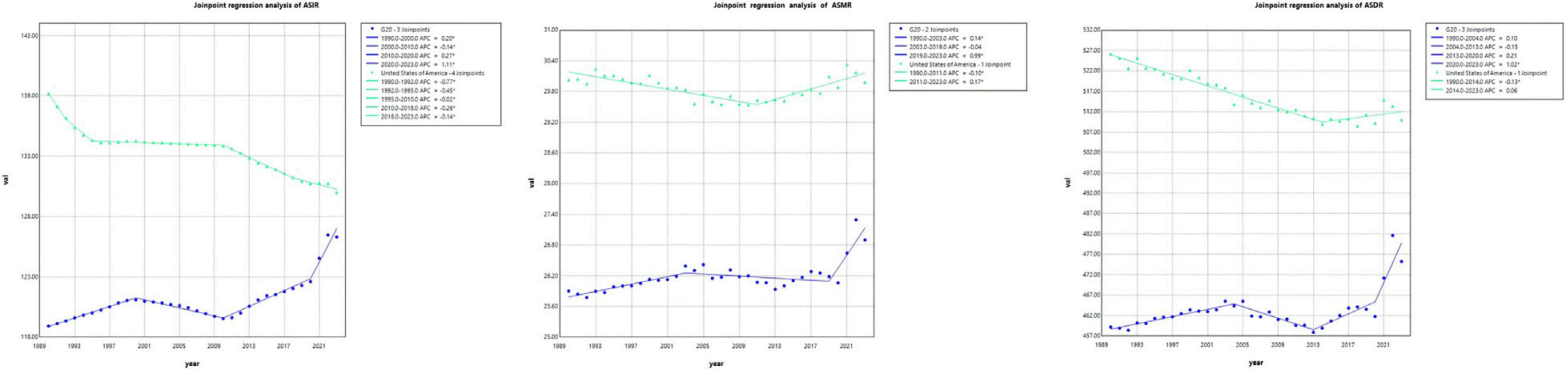
Joinpoint regression analysis of Age-Standardized Incidence, Mortality, and Disability-Adjusted Life Year Rates for Alzheimer’s disease and other dementias in the United States and Group of Twenty nations (Global Burden of Disease Study 2021, United States and G20 countries, 1990–2023).

**FIGURE 2:**
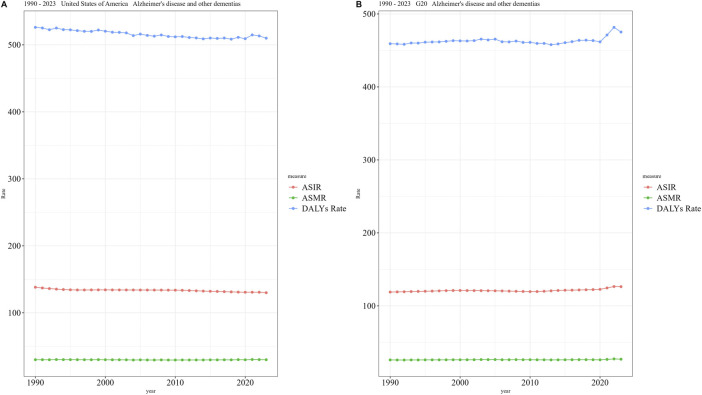
Comparison of trends in Age-Standardized Incidence, Mortality, and Disability-Adjusted Life Year Rates for Alzheimer’s disease and other dementias in the United States and Group of Twenty nations (United States and Group of Twenty nations, 1990–2023). **(A)** Trends in the United States (United States, 1990–2023). **(B)** Trends in the Group of Twenty nations (Global Burden of Disease Study 2021, United States and G20 countries, 1990–2023).

Figures 1, 2 delineate the overall trajectories of age-standardized incidence (ASIR), mortality (ASMR), and disability-adjusted life year (ASDR) rates for the United States and the G20 aggregate from 1990 to 2023. While the US exhibited a predominant downward trend in age-standardized rates, the G20 aggregate displayed a contrasting pattern characterized by greater volatility and a concerning recent deterioration.

Specifically, the US ASIR demonstrated a sustained monotonic decline, characterized by five distinct trend segments. The most precipitous reduction occurred between 1990 and 1995 (APC: -0.77 to −0.45, P < 0.05), after which the decline attenuated between 1995 and 2010, before resuming a significant downward momentum post-2010. Regarding US mortality, a critical trend reversal was observed: the ASMR significantly descended from 1990 to 2011 (APC = −0.10, P < 0.05), but inverted to a significant ascent from 2011 through 2023 (APC = 0.17, P < 0.05). Similarly, the US ASDR declined significantly from 1990 to 2014, but then stagnated into a statistically non-significant plateau.

In stark contrast, the G20 ASIR followed a fluctuating triphasic pattern: an ascent in 1990–2000, a descent in 2000–2010, and a re-entry into an upward trajectory after 2010 (APC = 0.27, P < 0.05). This recent rise culminated in a sharp acceleration between 2020 and 2023 (APC = 1.11, P < 0.05). G20 mortality and DALY rates, which remained relatively quiescent from 2003 to 2019, also exhibited substantial growth during the 2020–2023 interval (APC = 0.99 and 1.02, respectively; both P < 0.05). These contrasting patterns underscore a fundamental epidemiological bifurcation: while the US has largely achieved a plateau or decline in standardized rates, the G20 aggregate is witnessing a concerning resurgence in disease burden.

**FIGURE 3:**
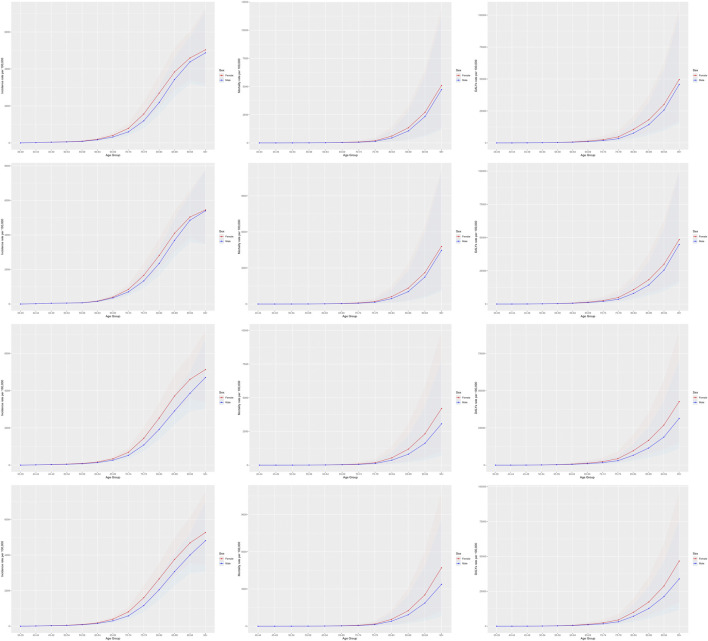
Comparative analysis of Age-Standardized Incidence, Mortality, and Disability-Adjusted Life Year Rates stratified by age group in the United States and Group of Twenty nations (Global Burden of Disease Study 2021, United States and G20 countries, 1990–2023).

**FIGURE 4:**
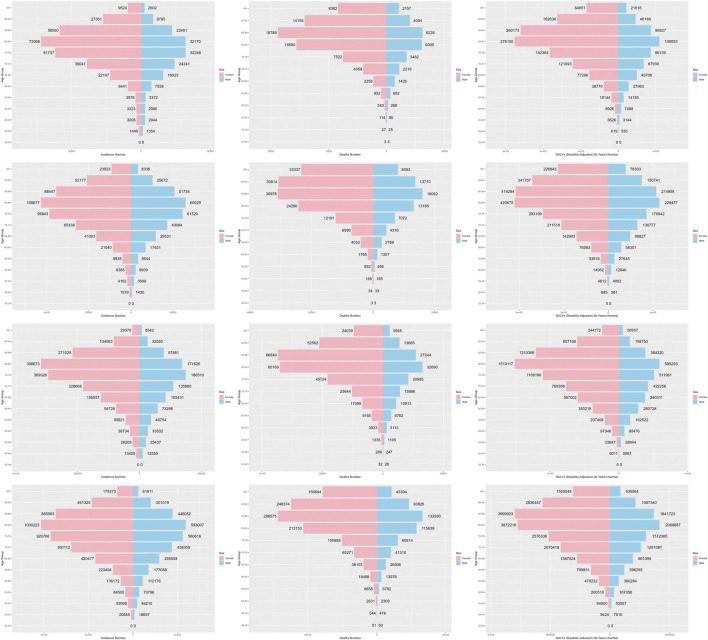
Comparative analysis of absolute Alzheimer’s disease and other dementias burden (incident cases, deaths, and Disability-Adjusted Life Years) stratified by age group in the United States and Group of Twenty nations (Global Burden of Disease Study 2021, United States and G20 countries, 1990–2023).

Figures 3, 4 delineate the age-standardized rates (Figure 3) and absolute metrics—incidence, mortality, and DALYs (Figure 4) across distinct age strata for the United States and G20 in 1990 and 2023. Absolute Counts regarding age-standardized rates, the trajectory consistently crested in the eldest cohort (≥95 years). However, in terms of absolute incident volume, ADOD cases were predominantly concentrated within the population aged 60 and above in both the US and G20, with a marked surge observed in the 75–89 age bracket. Mortality metrics mirrored this distribution. In 1990, the burden of DALYs in the US peaked within the 80–84 age group for both sexes, tapering off thereafter. Contemporaneously in G20, the male DALY peak aligned with the US (80–84 years), whereas the female burden peaked earlier, in the 75–79 age stratum, before declining. By 2023, the apex of incident cases had converged to the 80–84 age group across both regions and sexes. Crucially, a persistent sexual dimorphism was evident across both temporal milestones: incidence, mortality, and DALY rates were consistently elevated in females compared to males in both the US and G20. Supplementary Figure 1 illustrates the longitudinal evolution of caseloads and rates from 1990 to 2023. Sex-specific and age-standardized metrics exhibited temporal oscillations throughout the study period. Broadly, while the US ASIR demonstrated a downward trajectory, this did not translate to a reduction in absolute caseloads; conversely, total case numbers for both sexes trended upwards—a phenomenon driven by demographic shifts. The female ASIR remained persistently elevated above that of males, a pattern consistent with G20.

### Projections of Alzheimer’s disease and other dementias incidence, mortality, and disability-adjusted life years (DALYs) in the United States and G20 nations, 2024–2040

Time-series forecasting utilizing ARIMA models elucidates a significant bifurcation in the epidemiological profiles of Alzheimer’s disease and other dementias between the United States and G20 over the approaching seventeen-year horizon (Table 2). Optimal model parameterization (validated via AIC and BIC criteria; see Supplementary Table S1) indicates that, despite ubiquitous global aging pressures, the evolution of disease burden in these two territories will manifest markedly distinct trajectories. For the United States, a substantial amelioration in incidence risk is juxtaposed against a stubborn persistence in mortality and disability burdens. Both male and female age-standardized incidence rates (ASIR) are projected to enter a descending channel. Specifically, the male ASIR is anticipated to recede gradually from 116.15 per 100,000 in 2024 to 107.04 per 100,000 by 2040 (a reduction of approximately 7.8%); the magnitude of improvement is projected to be even more pronounced among females, with the rate declining from 138.62 per 100,000 to 126.83 per 100,000, a decrease of 8.5%. In contrast to the significant decline in incidence, the age-standardized mortality rate (ASMR) shows no appreciable reduction. The US male ASMR is expected to maintain a level of approximately 26.05 per 100,000 (a marginal decrease of 0.04%), while the female ASMR is projected to fluctuate only slightly from 32.87 per 100,000 to 32.83 per 100,000 (−0.12%), exhibiting characteristic plateau dynamics (Supplementary Figure 2). Similarly, the rate of disability-adjusted life years (DALYs) shows no significant improvement, with female projections stabilizing at 566.00 per 100,000 and males showing only a narrow contraction of 1.3%.

**TABLE 2:** Projected values for Alzheimer’s disease and other dementias incidence, mortality, and disability-adjusted life years (Global Burden of Disease Study 2021, United States and G20 countries, 1990–2023).

Location	Year	Age-standardized incidence rate, per 100,000 people		Age-standardizedmortalityrate, per 100,000 people		Age-standardized DALYs rate, per 100,000 people	
​	​	Male	Female	Male	Female	Male	Female
USA	2024	116.15	138.62	26.05	32.87	437.68	566.00
	2040	107.04	126.83	26.04	32.83	432.10	566.00
% Change	​	−7.84%	−8.51%	−0.04%	−0.12%	−1.27%	0.00%
G20	2024	109	139.52	22.05	30.02	390.90	535.59
	2040	112.46	144.1	22.98	30.02	404.22	535.59
% Change	​	​	3.17%	3.28%	4.22%	0.00%	3.41%

In stark contrast to the ameliorative trend in the US, the G20 as an aggregate face the severe challenge of a burden resurgence, particularly within the male population. Neither males nor females in G20 exhibit signs of decline in ASIR; conversely, a moderate accretion of approximately 3% is observed. By 2040, male and female incidence rates are projected to climb to 112.46 per 100,000 and 144.10 per 100,000, respectively, signaling an accumulation of potential public health pressure. The predictive models specifically highlight the vulnerability of the male demographic. The ASMR and age-standardized DALY rate (ASDR) for G20 males are projected to increase by 4.2% and 3.4%, reaching 22.98 per 100,000 and 404.22 per 100,000, respectively, by 2040. By comparison, female mortality and disability metrics demonstrate pronounced inertia, projected to sustain current levels over the next two decades with a lack of ameliorative momentum.

### Attributable risk factors for Alzheimer’s disease and other dementias DALYs in G20 nations

Supplementary Figure 3 illustrates the proportional contribution of attributable risk factors (high fasting plasma glucose and smoking) to Alzheimer’s disease and other dementias DALYs across G20 in 1990 and 2023. Our analysis revealed substantial heterogeneity among G20 regarding the proportion of DALYs attributable to high fasting plasma glucose and smoking. Notably, the disparity in DALYs attributable to high fasting plasma glucose was particularly pronounced.

In 1990, the three nations exhibiting the highest proportion of DALYs attributable to high fasting plasma glucose were the United Kingdom (21.1%), Mexico (21.0%), and Saudi Arabia (20.8%). The United States occupied a relatively intermediate position among G20, with a proportion of 16.5%. Concurrently, the top three G20 for DALYs attributable to smoking were China (7.6%), the United Kingdom (7.5%), and Canada (7.5%), whereas the attributable proportion in the US stood at 5.4%.

By 2023, a pervasive upward trend was observed in the proportion of DALYs attributable to high fasting plasma glucose. The nations with the highest burden shifted to Saudi Arabia (26.2%), the United Kingdom (23.4%), and Japan (21.4%), with the US recording 20.7%. Compared to 1990, this proportion in the US rose by 4.2 percentage points, while Saudi Arabia experienced an increase of 5.4 percentage points. Conversely, the proportion of DALYs attributable to smoking demonstrated a marked recession. In 2023, the highest proportions were observed in China (5.9%), Canada (5.0%), and the United States (4.9%). Notably, the smoking-attributable proportion in the United Kingdom plummeted from 7.5% in 1990 to 4.1%, and the majority of G20 witnessed varying degrees of decline in smoking-attributable burdens during the observation period.

## Discussion

Departing from single-nation analyses or outdated projections, this study integrates comparative trend decomposition (Joinpoint) with predictive modeling (ARIMA) based on the updated GBD 2023 metrics [11, 12]. This dual-modeling approach provides a robust mechanism to validate the structural divergence in disease trajectories between the US and G20 economies. Our analysis crystallizes a novel paradigm: the “US-G20 divergence.” We conclude that the US burden is no longer driven by a failure of medical intervention (as rates are dropping), but by demographic inertia and metabolic vulnerability [13, 14]. The identification of High Fasting Plasma Glucose as a rapidly escalating attributable risk factor—surpassing smoking—constitutes a crucial pivot point in ADOD epidemiology [15]. This establishes a critical priority for future policy: moving beyond generic “healthy aging” to targeted geriatric metabolic management [16].

### Interpretation of overall trends and driving factors

Our investigation elucidates that from 1990 to 2023, although the absolute caseload and mortality of Alzheimer’s disease and other dementias (ADOD) in the United States escalated significantly due to population aging, the aggregate growth of DALYs remained relatively contained. Concurrently, the age-standardized incidence (ASIR), mortality (ASMR), and DALY (ASDR) rates for the total US population exhibited a predominant downward trajectory over the past three decades. This is consistent with the previous research results on the “compression of incidence rate of dementia” in high-income countries [17]. Based on our ARIMA forecasting models, the ASIR, ASMR, and ASDR for ADOD in the US are projected to sustain a steady decline or stabilize into a plateau phase in the coming years.

We attribute this volume increase, rate decrease phenomenon to a confluence of driving factors. First, improvements in socioeconomic status and educational attainment across the US population have played a pivotal role. The significant rise in higher education prevalence over recent decades has likely bolstered “cognitive reserve,” enabling the brain to better tolerate neuropathological changes without manifesting clinical symptoms, thereby explaining the observed decline in standardized rates [18, 19]. Second, the spillover effects of cardiovascular health strategies on ADOD burden warrant consideration. Since the early 21st century, the US healthcare system has widely implemented screening [20] and intervention programs targeting hypertension and hyperlipidemia [21]. Given the intimate link between vascular and cerebral health, these vascular protection measures have significantly reduced the risk of mixed dementia and vascular cognitive impairment, indirectly enhancing ADOD prevention and control [22, 23]. Third, the reduction in incidence and mortality may also be ascribed to positive lifestyle shifts [24, 25]. Since 2005, the implementation of stringent tobacco control strategies in the US has effectively curbed the prevalence of smoking—a key neurotoxic risk factor—making a substantial contribution to mitigating dementia risk at the population level [26, 27].

### The paradox of high levels and international disparities

Despite the downward trend in standardized rates over the past 30 years, the absolute disease burden in the US remains heavy due to its large population base and deep aging. Notably, we observed that the ASIR, ASMR, and ASDR for ADOD in the US in both 1990 and 2023 remained significantly higher than the G20 average. What underpins this disparity? A reason may lie indetection biasand competing risks [28, 29]. Compared to some emerging economies within the G20, the US possesses a more robust dementia surveillance system and higher healthcare utilization rates. Furthermore, insurance coverage facilitates widespread cognitive screening, leading to a high detection rate of cases [30], whereas many G20 may suffer from significant underdiagnosis or underestimation [31].

Another critical factor is the divergence in risk factor control. Our study reveals that in 2023, the proportion of ADOD DALYs attributable to high fasting plasma glucose rose universally across G20, with the US remaining at an elevated level. While the US has achieved success in tobacco control, it faces severe challenges in metabolic health. Our data indicate that by 2023, high fasting plasma glucose had superseded smoking as a primary attributable risk factor. This underscores the necessity of strengthening metabolic health management, for instance, through policy interventions to reduce ultra-processed food consumption, the promotion of Mediterranean dietary patterns, and enhancing public awareness of the metabolic-cognitivenexus. Existing research suggests that insulin resistance and chronic hyperglycemia can exacerbate amyloid-beta deposition and tau phosphorylation, thereby accelerating ADOD pathology.

We acknowledge that the attributable fraction of these two specific risk factors is relatively modest (approximately 4% according to the Lancet Commission estimates). However, our selection was strategically driven by their representation of two distinct modifiable pathways—behavioral (smoking) and metabolic (high fasting plasma glucose). Importantly, our findings reveal a critical epidemiological “pivot”: the contribution of smoking to the ADOD burden is waning globally, whereas the contribution of high fasting plasma glucose is surging. This trend, even at a modest attributable level, signals a fundamental shift in future public health priorities—moving beyond generic risk reduction toward targeted geriatric metabolic management, specifically to preserve the gains achieved through decades of tobacco control.

The entry of the “Baby Boomer” generation into high-risk age brackets: Over the past three decades, the large U.S. “Baby Boomer” cohort (born between 1946 and 1964) has progressively entered older adulthood [32]. This massive population group moving into the high-risk age zone of 65 and above has directly driven up the total case count. Even though age-standardized rates aim to adjust for the influence of age structure, this distinct demographic shockwave effect remains strikingly evident in the absolute numbers.

### Short-term fluctuations and demographic characteristics

Results from the Joinpoint regression model indicate that while the overall trend for US ADOD ASIR and ASMR was downward from 1990 to 2023, there were intervals of fluctuation or transient increase. The root causes of this phenomenon remain not fully elucidated but may be linked to changes in diagnostic criteria and coding rules. Some scholars posit that these trends might relate to transitions in ICD coding systems [33] and heightened public awareness following the signing of the National Alzheimer’s Project Act (NAPA) [34], which could lead to a short-term artificial increase in reported cases.

The burden of ADOD exhibits clear variation by sex and age. Consistent with the broader pattern observed in the GBD study and other reports, our analysis confirms that at the age-standardized level, the incidence and mortality rates for ADOD are indeed higher in females than in males. This observed disparity may be attributed to several interconnected factors. First, the longer average life expectancy of females results in a larger population surviving into the highest-risk age groups where ADOD prevalence escalates. Second, biological mechanisms, such as the decline of neuroprotective estrogen after menopause, are hypothesized to contribute to elevated female risk in later life. Importantly, while the absolute burden is higher in females, the trend analysis within the G20 indicates a concerning relative deterioration in the male burden over time. This shift may be driven by the cumulative impact of higher exposure among males to modifiable risk factors such as smoking, alcohol use, and cardiovascular disease, which are becoming more significant determinants of disease trajectory.

### Policy implications and limitations

These findings suggest that formulating precision prevention and control policies based on sex and regional specificities is crucial for curbing the spread of ADOD. As the US population ages, the disease burden is increasingly concentrated in the 75+ age demographic [8, 35]. Given that older adults often present with multimorbidity, the clinical management of ADOD becomes more complex [35, 36]. Therefore, strengthening comprehensive chronic disease management for the older adult(s) is vital for mitigating future burdens. Simultaneously, for the US and developed economies, the focus should shift towards “refined management of the oldest-old” and regulation of the “metabolic-cognitive axis.” With the peak incidence shifting to those aged 95 and above, healthcare systems must establish integrated care models adapted to multimorbidity to address the challenge of co-occurring diabetes and dementia [37]. Second, for emerging economies within the G20, particularly those facing a rebound in male incidence, there is an urgent need to break the barrier of “prioritizing treatment over prevention” and establish sex-specific intervention strategies, focusing on curbing risk accumulation among males during periods of lifestyle transition [38]. Finally, the global health agenda should strive to eliminate the diagnostic divide by promoting low-cost biomarker testing, transforming invisible disease burdens into actionable clinical data [39, 40]. Only by addressing the diagnostic disparities behind the “paradox of high levels” can we truly achieve the ambitious goal of mitigating the global burden of ADOD by 2040.

It is important to note the limitations of our study. First, the heterogeneity of disease burden data sources remains a persistent challenge in GBD research. The quality of primary cause-of-death registration data in some G20 varies, which may not fully reflect the true epidemiological characteristics of the disease [41]. Second, due to the lack of subtype classification data based on biomarkers in the GBD database, we could not differentiate the burden of ADOD from other dementia types in terms of anatomy and pathology. Clinical studies indicate that mixed dementia is more common in the older adult(s), which may lead to biases in attribution analysis [42]. Finally, as the ARIMA model extrapolates based on historical data, it may not fully capture the potential interference of the long-term neurological impacts of the COVID-19 pandemic [43]. Nevertheless, our primary findings are generally consistent with previous multinational cohort studies, providing a solid empirical basis for global health policy formulation.

### Conclusion

Based on the GBD 2023 data framework, this study conducted a systematic assessment and prospective forecast of the epidemiological burden and future trends of Alzheimer’s disease and other dementias in the United States and G20. Retrospectively, over the past three decades, the age-standardized incidence, mortality, and DALY rates of ADOD in the US have shown positive improvement, driven by the continuous optimization of public health prevention strategies, significant enhancements in medical care, and steady improvements in socioeconomic conditions. However, despite these favorable trends, a significant level gap persists between the US and its G20 peers, with the absolute disease burden in the US remaining high. In particular, our attribution analysis highlights that the DALY burden attributable to high fasting plasma glucose and smoking in the US remains significantly above the G20 average, underscoring the urgency of metabolic risk management within the current prevention system. Looking forward, the US urgently requires more targeted precision prevention strategies. Policymakers should transition fromone-size-fits-allintervention models to individualized management based on risk factors (with a focus on metabolic health) and demographic characteristics (covering males, females, and the oldest-old). Furthermore, mobilizing societal health engagement, elevating clinical care standards for neurodegenerative diseases, and accelerating the development of frontier diagnostic and therapeutic technologies for ADOD (such as biomarker screening and disease-modifying therapies) will be key pathways to further reducing age-standardized incidence rates and alleviating the societal burden.

## Data Availability

The data used are publicly available online on the website of the Institute for Health Metrics and Evaluation (IHME) (https://ghdx.healthdata.org/gbd-2023).

## Supplementary material

SUPPLEMENTARY FIGURE 1 Case numbers and Age-Standardized Incidence, Mortality, and Disability-Adjusted Life Year Rates for males and females across age groups in the United States and Group of Twenty nations (United States and Group of Twenty nations, 1990–2023).

SUPPLEMENTARY FIGURE 2 Longitudinal trends in Age-Standardized Incidence, Mortality, and Disability-Adjusted Life Year Rates of Alzheimer’s disease and other dementias in the United States and Group of Twenty nations, with forecasts for the 2024–2040 period (United States and Group of Twenty nations, 1990–2040).

SUPPLEMENTARY FIGURE 3 Comparison of the proportional contribution of attributable risk factors to Alzheimer’s disease and other dementias Disability-Adjusted Life Years in the Group of Twenty nations (Group of Twenty nations, 1990 and 2023).
